# *Azolla spp* and *Hermetia illucens* Meals as Main Protein Sources for Rabbit Nutrition: Impact on Feed Quality, Growth Performance, and Meat Quality

**DOI:** 10.1016/j.cdnut.2025.107595

**Published:** 2025-11-01

**Authors:** Eyitayo Azaratou Ogbon, Arnette Balè, Carline Santos, Louckman Monra Seïdou, Daniel Dzepe, Justin G Behanzin, Rousseau Djouaka

**Affiliations:** 1 International Institute of Tropical Agriculture, Platform AgroEcoHealth, Cotonou, Benin; 2 Faculty of Science and Technology, University of Abomey-Calavi, Abomey-Calavi, Benin

**Keywords:** *Azolla spp*, *Hermetia illucens*, protein digestibility, rabbit growth, rabbit meat quality

## Abstract

**Background:**

Good-quality feed is essential for successful livestock production and for ensuring the distribution of high-quality, safe animal products.

**Objectives:**

This study evaluated the impact of using Azolla and black soldier fly (BSF) larvae meals as the main protein ingredients in formulated rabbit feed on the quality of the feed and the meat produced.

**Methods:**

Three isocaloric diets were formulated: Az_diet (Azolla meal as the main protein source), BSF_diet (BSF larvae meal as the main protein source), and SM_diet (soybean meal as the main protein source). A palatability test was carried out on 5 feeds: Az_diet, BSF_diet, SM_diet, live BSF larvae, and fresh Azolla leaves. These feeds were offered to 5 rabbits (aged 12 wk) for 10 d in a free-choice test. The growth test was carried out on 36 rabbits aged 5–6 wk. The rabbits were divided into 3 groups of 12 and fed 1 of the 3 diets (Az_diet, BSF_diet, or SM_diet) ad libitum for 42 d. The measured parameters were feed quality and health quality of rabbit meat.

**Results:**

The result test showed that the SM_diet was the most palatable for the rabbits, representing 82.03% of the feed consumed during the free-choice feeding test. No mortality or significant differences in feed intake were recorded for the 3 treatments. The BSF_diet treatment resulted in significantly higher weight gain (1012.5 ± 170.13 g). The Az_diet had the highest protein digestibility (80.39 ± 2.08%). The nutritional composition of the hind leg meat of rabbits was similar for the 3 diets. No lead or cadmium was detected in the hind leg meat of rabbits fed the 3 diets.

**Conclusions:**

In this study, using Azolla and BSF larvae meal as the main source of dietary protein did not negatively affect feed quality, rabbit growth, or rabbit meat quality.

## Introduction

The rapid increase in the world population is leading to increased demand for food, particularly for dietary protein [1]. To meet the demand for animal protein, the production of short-cycle animals such as rabbits appears to be a promising alternative [2]. Consequently, In Africa, rabbit meat production has increased by 23.5%. Indeed, rabbit meat is highly prized because of its high-quality protein, low fat, and low cholesterol content [2]. However, several factors hinder the development of rabbit farming. One such factor is the cost of feed, particularly the cost of protein ingredients essential for the healthy growth of animals [3]. Exploring and developing of alternative protein ingredients could lead to greater feed autonomy for this sector [3].

*Azolla spp* (Azolla) and *Hermetia illucens* [black soldier fly (BSF)] are new recently promoted sustainable protein ingredients for animal feed. Indeed, BSF larvae (BSFL) can be raised from organic waste [4]. They contain ∼35%–42% crude protein on a dry matter (DM) basis. This makes them comparable with soybean meal in terms of both biological value and amino acid profile [5]. The most common macrominerals found in BSFL are calcium (Ca), phosphorus (P), potassium (K), and magnesium (Mg), as well as trace elements such as zinc (Zn), copper (Cu), selenium (Se), and iron (Fe). The concentrations of these minerals vary considerably depending on the rearing substrate used for the larvae, but may include concentrations of phosphorus between 0.18% and 1.11% of DM, potassium between 0.16% and 4.41%, magnesium between 0.01% and 1.06%, and Zn between 0.002% and 0.041% [6,7]. As for Azolla, it is a symbiotic aquatic plant associated with nitrogen-fixing algae. Azolla contains 20.4%–28.5% crude protein on a DM basis. Therefore, Azolla and BSFL are 2 new environmentally friendly protein ingredients that do not compete with human consumption. However, any new ingredient introduced into a feed formula must maintain or even improve the quality of the formulated feed. A good-quality feed, apart from meeting all the animal's nutritional needs, must be palatable, digestible, and healthy [8].

Many factors can influence the acceptability and feed preference of rabbits, such as olfactory appeal, taste, size and texture, and the nature of the ingredients [3]. A feed preference test is therefore recommended for the selection of ingredients that will maximize the efficiency of rabbit production. Although there are increasing reports of Azolla and BSFL meals being incorporated into rabbit diets, very few of these involve free-choice trials with pellets formulated using these ingredients [[9], [10], [11], [12]]. Furthermore, to our knowledge, there is no information on the digestibility of BSFL protein in rabbits. Additionally, compared with other monogastric animals, the literature provides limited information on the digestibility of Azolla protein in rabbits. Most research on Azolla and BSFL protein digestibility has focused on other species of animal. Bhatt et al. [13] demonstrated that the addition of Azolla to calf and goat diets improved crude protein digestibility (CDP). On the other hand, Das et al. [14] and Abou El-Fadel [15] observed that protein digestibility decreased significantly as the proportion of Azolla in lamb diets increased. Studies have shown that BSFL accelerated the growth performance and nutrient digestibility of poultry. Therefore, protein digestibility varies not only depending on the animal species, but also on the incorporation rate of the protein source. Further research is needed to better understand the absorption percentage of Azolla and BSFL proteins in the rabbit digestive tract.

Regarding food safety, previous studies have demonstrated that Azolla and BSFL can bioaccumulate heavy metals from their rearing substrates (water and organic waste, respectively) [[16], [17], [18]] According to Purschke et al. [19], BSFL can bioaccumulate Cd and lead (Pb) with accumulation factors of 9 and 2, respectively. Azolla can absorb concentrations of 10,000 mg/L, 1990 mg/L, 9000 mg/L, and 650 mg/L, respectively, for Cd, Cr, Cu, and Ni [20,21]. Contamination of food with heavy metals poses a significant public health risk [22,23]. Furthermore, WHO [1] reported that Pb and Cd are among the 10 most hazardous chemicals of greatest public health concern. These heavy metals can cause serious illness and even death in humans and animals [24,25]. Previous studies have reported the accumulation of Pb and Cd in rabbit organs. According to Szkoda et al. [26], the mean Pb value in rabbit meat in Poland was 0.03 mg/kg (wt:wt) and 0.17 mg/kg (wt:wt) in rabbit liver. An analysis of rabbit meat samples sold in Nigeria revealed the average levels of 0.04 ± 0.003 mg/kg and 0.02 mg/kg for Pb and Cd, respectively. Although the values found in these studies were below the limit for human consumption, confirmation of the potential bioaccumulation of these heavy metals in rabbit meat highlights the importance of scrutinizing rabbit feed more closely. Given the above, it seems important to understand the effect of Azolla and BSFL meals on the quality of formulated rabbit feed and rabbit meat. Therefore, the objective of this work was to evaluate palatability and protein digestibility of pellets made with Azolla and BSFL meals as the main protein ingredients, as well as their impact of these pellets on the nutritional and health quality of rabbit meat.

## Methods

### Ethical approval

This study received approval No. 005-2025/EPAC/LARBA/URMA/CE/R from the Ethical Committee of Research Unit on Communicable Diseases (URMAT) at the Polytechnic School of Abomey-Calavi/University of Abomey-Calavi/Benin.

### Experimental site

Rabbit breeding took place in the greenhouse at the International Institute of Tropical Agriculture (IITA) in Benin. IITA Benin is located in the commune of Abomey-Calavi in the Atlantic department (6°28'N, 2°21'E, 15 m above sea level), specifically in Tankpè, where the average ambient temperature ranges from 29°C to 34°C.

### Experimental diets

Three diets were formulated during this study, in accordance with the proportions presented in Table 1. These were Az_diet, BSF_diet, and SM_diet. The SM_diet was formulated according to the composition of a commercial diet commonly used in rabbit breeding facilities in Benin, as described by Alabi et al. [27,28]. This diet used soybean meal as the main source of protein. The Az_diet and BSF_diet were diets in which the soybean meal had been replaced, respectively, with *Azolla spp*. meal and BSFL meal. Fresh *Azolla spp*. leaves and BSFL were collected from the greenhouse at the AgroEcoHealth platform of IITA Benin. These were then dried in a dehydrator at 50°C for 12 h for Azolla and 48 h for BSFL. The dried Azolla leaves (AZL) and dried BSFL were ground separately using a mixer. The ingredients and nutritional composition of the experimental diets are presented in Table 1.

**TABLE 1:** Proportion of ingredients of Az_diet, BSF_diet, and SM_diet expressed on a dry matter basis

Ingredients (%)	SM_diet^1^	Az_diet^2^	BSF_diet^3^
Soybean meal	10	0	0
Azolla meal	0	10	0
BSF larvae meal	0	0	10
Corn	4	8	4
Cottonseed meal	4	8	4
Wheat bran	34.5	34.50	31
Palm kernel meal	21	21	21.50
Rice bran	20.5	16.50	21.50
Oyster shell	2.5	2.50	2.50
Salt	0.25	0.25	0.25
Vitamin	0.25	0.25	0.25
Total	100	100	100

Abbreviations: Az_diet, Azolla meal as the main source of protein; BSF, black soldier fly; BSF_diet, BSF larva meal as the main source of protein; SM_diet, soybean meal as the main source of protein.

1 Soybean meal as the main source of protein.

2 Azolla meal as the main source of protein.

3 BSF larva meal as the main source of protein.

### Prophylaxis

The veterinary drugs used during the experiment were Sulfadimidin PG (PharmaGal) and Total Amin (LAPROVET). Sulfadimidin PG is a veterinary product used to prevent coccidiosis. Total Amin is a vitamin supplement used to prevent stress and revitalize the rabbits.

### Experimental design

**FIGURE 1:**
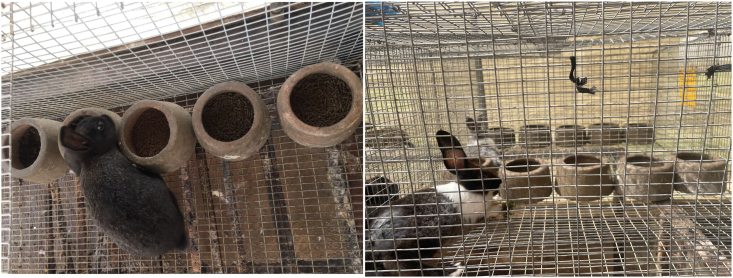
Arrangement of feeders in the cage during the free feed choice test.

A total of 41 Hyla breed rabbits were used in this experiment. Of these, 36 weaned rabbits aged 5–6 wk and weighing 350–370 g (sex ratio 1:1) were used for the feeding trial, and 5 adult rabbits aged 12 wk (3 males and 2 females) were used for the diet palatability test. The rabbits were housed individually. Five diets were tested: Az_diet, BSF_diet, SM_diet, live BSFL, and fresh AZL. Feed palatability was determined by the rabbits' free choice of the 5 test feeds, according to the method described by Aldrich and Koppel [29]. For this purpose, each rabbit received 5 identical feeders containing a different diet each morning. Each feeder contained 50 g of test diet, with the feeders mounted on opposite sides of the cage (Figure 1). The position of the feeders in the cage was changed daily. The behavior of each rabbit toward the diet was observed for 1 h and the first diet chosen was noted. The remainder in each feeder was also weighed after 1 h (every day, at the end of the test). The test lasted 1 h/d for 10 d.

For the growth test, the rabbits were randomly divided into batches of 12 per treatment. There were 3 treatments in total: the control treatment (rabbits fed the SM_diet); treatment A (rabbits fed the Az_diet); and treatment B (rabbits fed the BSF_diet). The rabbits were housed 4 per cage. These galvanized metal cages measured 78 cm in length, 54 cm in width, and 40 cm in height. Each cage was equipped with a feeder and a drinker. The rabbits were acclimatized to the experimental diet for 7 d. Feed and water were then provided to the rabbits ad libitum during the experiment. The experiment lasted 42 d to allow them to reach the recommended kill age of 9–13 wk [30]. The rabbits were weighed weekly. The remaining feed was weighed every morning. To assess the rabbits' CDP, feces were sampled during the final 7 d of the experiment [31]. Feces were collected every morning into small zip-lock bags using forceps. The remaining feces were removed from the cage every day to avoid mixing with the following day's feces. The samples were stored in a freezer at −20°C until the day of analysis.

### Kill procedure and rabbit meat sampling

At the end of the feeding trial, 3 rabbits (2 males and 1 female) from each treatment group were randomly selected, weighed, fasted for 12 h and killed. They were killed using the Blasco and Ouhayoun [32] method. The rabbits were stunned by a sharp blow to the base of the neck, resulting in instant death. They were then killed by cutting through the jugular vein and carotid arteries below the jaw. After bleeding, the entire carcass was weighed. Then, the liver, kidneys, and entire digestive tract were dissected and weighed, as were the carcass muscles. Fifty g of meat from the hind leg were taken for analysis.

### Physicochemical analysis

DM content was measured by oven drying at 104°C for 24 h. Crude protein (in diet, meat, and feces) was measured using the Kjeldahl method [33]. The crude lipid content of meat samples was measured using the Soxhlet method with petroleum ether [34]. Combustion in an adiabatic calorimeter (C5000, IKA-Werke) is the method used to measure the gross energy of diets. For amino acid analysis, sulfur-containing amino acids were extracted by oxidation with peroxyformic acid followed by low-temperature hydrolysis for 16 h. Lysine was extracted by hydrolysis with hydrochloric acid at 110°C for 23 h. The hydrolyzate was dissolved in citrate buffer at pH 2.2. The extracted amino acids were analyzed using an AAA-400 amino acid analyzer (INGOS Ltd.).

Minerals extraction and quantification were performed for diets and meat samples. Minerals extraction was performed according to the protocol described by Ogbon et al. [35]. The samples were oven-dried at 105°C until they reached a constant weight. The dried samples were then ground into a powder using a mortar and pestle. The powder obtained from each sample was then sieved using a 2-mm mesh sieve. After sieving, 2 g of the sieved powder from each sample was weighed and digested with 20 mL of a mixture of concentrated HNO_3_ and H_2_SO_4_ (3:1 vol:vol). Digestion was performed in a water bath at 95°C until a clear solution was obtained. After cooling, each digested sample was filtered using Whatman filter paper. The samples were then reconstituted in 30 mL of an aqueous solution containing 0.5% hydrochloric acid and 2% nitric acid to stabilize the elements as an ionic solution. The samples were stored at 4°C for subsequent analysis. Cd, Cu, Pb, and Zn were measured using a Metalyser HM 3000 (Trace2O). Ca, Fe, and P were quantified using an atomic absorption spectrophotometer equipped with a VARIANT flame and Spectra A110 software.

### Calculation of parameters

The palatability of each tested diet was defined as the quantity of the tested diet consumed expressed as a percentage of the total consumption of the 5 diets [27]:

⁃ $\text{Palatability}=\frac{\text{Feed}\;\text{consumed}\;\text{per}\;\text{diet}}{\text{Total}\;\text{feed}\;\text{consumed}}\times 100\;$ (1)

The biological parameters used to evaluate the efficacy of the diets tested on rabbits were calculated according to the following equations used by Volek et al. [36]:

⁃ Estimated total feed intake (FI):

(2) $$\text{FI}\;\left(g\right)=\begin{array}{ccc} \text{feed}\;\text{given}\; & - & \text{remaining}\;\text{feed}\; \end{array}$$

⁃ Weight gain (WG):

(3) $$\text{WG}\;\left(g\right)=\left(\begin{array}{ccc} \text{final} & - & \text{initial} \end{array}\right)\;\text{live}\;\text{weight}$$

⁃ Feed conversion ratio (FCR):

(4) $$\text{FCR}=\frac{\text{estimated}\;\text{feed}\;\text{intake}}{\text{live}\;\text{weight}\;\text{gain}}$$

⁃ Crude protein digestibility (CDP):

(5) $$\text{CDP}=\frac{\text{feed}\;\text{protein}\;\text{content}-\text{feces}\;\text{protein}\;\text{content}\;}{\text{feed}\;\text{protein}\;\text{content}}\times 100$$

⁃ $\text{Carcass}\left(\%\right)=\frac{\text{Carcass}\;\text{weight}}{\text{Rabbit}\;\text{live}\;\text{weight}}\times 100\;$(6)

The collected data were analysed using R (version 4.0.2). ANOVA one-way was used to compare mean at significance level 5% and Tukey HDS was used to seperate the means.

## Results

### Impact of Azolla and BSFL meals on the quality of formulated rabbit diets

#### Impact of Azolla and BSFL meals on the palatability of formulated rabbit diets

**FIGURE 2:**
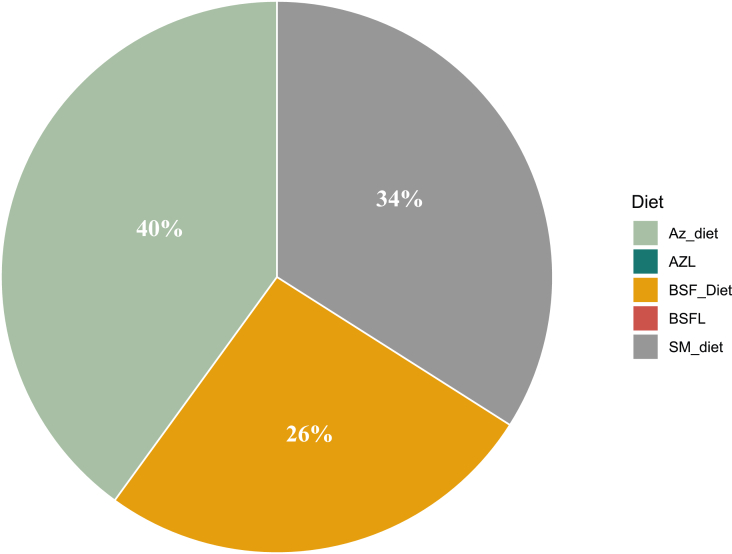
Percentage of diet chosen first by rabbits; Az_diet (Azolla meal as the main source of protein), BSF_diet (BSF larva meal as the main source of protein), SM_diet (soybean meal as the main source of protein), BSFL (live black soldier fly larvae), and AZL (fresh *Azolla spp*. leaves).

**FIGURE 3:**
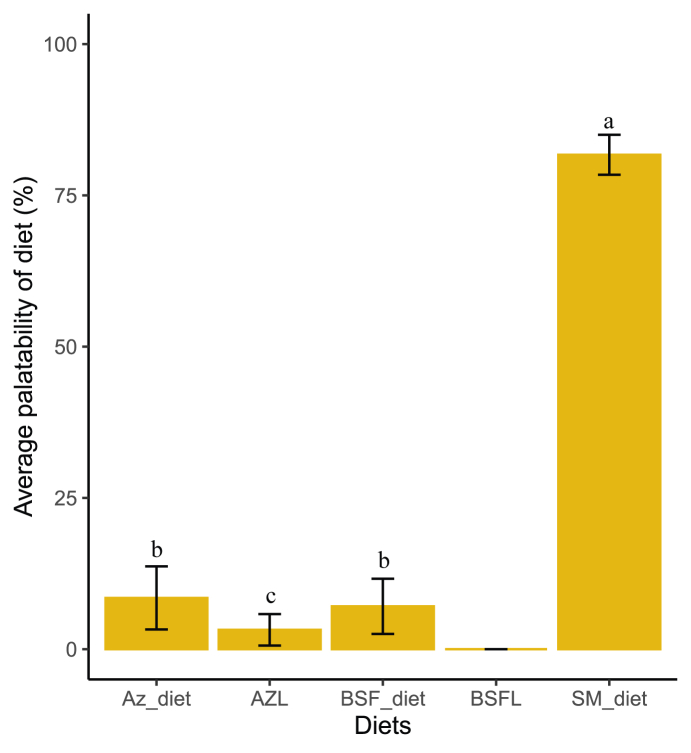
Palatability of the formulated rabbit diets. Az_diet (Azolla meal as the main source of protein), BSF_diet (BSF larva meal as the main source of protein), SM_diet (soybean meal as the main source of protein), BSFL (live black soldier fly larvae), and AZL (fresh *Azolla spp*. leaves). Averages followed by different letters differ significantly at the 5% level.

Palatability test showed that the Az_diet was the first choice of feed for 40% of the rabbits studied, followed by the SM_diet (34%) and the BSF_diet (26%) (Figure 2). There was no significant difference between these values. AZL and live BSFL were not the first choice of feed for any of the rabbits studied. The average palatability of each diet during the test period is presented in Figure 3. In all, 81.71 ± 3.3% of the feed consumed was SM_diet, 8.47 ± 5.21% was Az_diet, 7.08 ± 4.56% was BSF_diet, and 3.20 ± 2.61% was AZL. There was no significant difference between the palatability of BSF_diet and Az_diet. Only the BSFL (live BSF larvae) was not consumed among the 5 diets presented to the rabbits.

#### Impact of Azolla and BSFL meals on nutritional composition and heavy metal content of the formulated rabbit diets

The nutritional composition and heavy metal content of the formulated diets, expressed on a DM basis, are presented in Table 2 [37,38]. There were no significant differences in the total energy, lipid, or protein content of the 3 formulated diets. The 2 amino acids analyzed were methionine and lysine. The Az_diet had the lowest lysine and methionine content. Conversely, the BSF_diet had the highest lysine and methionine content. There was no significant difference in the methionine content of the BSF_diet and the SM_diet (soybean meal as the main protein source). The minerals targeted in this study were essential elements including Cu, Zn, phosphorous (P), Fe as well as nonessential elements such as Cd and Pb. With the exception of Pb, which was only detected in the Az_diet (Azolla meal as the main protein source), all other minerals were present in all 3 experimental diets. Additionally, the BSF_diet (with BSFL meal as the main protein source) had the highest Zn content, whereas the Az_diet had the highest Cu, P, Fe, Cd, and Pb content (Table 2).

**TABLE 2:** Nutritional composition and heavy metal content of Az_diet, BSF_diet, and SM_diet expressed on a dry matter basis

Nutritional composition		SM_diet^1^	Az_diet^2^	BSF_diet^3^	*P* value
Dry matter (%)		86.67 ± 1.15	87.53 ± 1.36	87.8 ± 0.92	0546
Crude protein (%)		16.71 ± 0.19	16.88 ± 0.01	16.75 ± 0.25	0.684
Crude fat (%)		5.70 ± 0.02	5.64 ± 0.04	5.65 ± 0.02	0.0822
Gross energy (MJ/kg)		15.23 ± 0.05	15.24 ± 0.1	15.26 ± 0.03	0.5775
Lysine (g/kg)		7.35 ± 0.09b	6.44 ± 0.13c	8.71 ± 0.07a	3.61e-07
Methionine (g/kg)		4.01 ± 0.04a	2.31 ± 0.09b	3.9 ± 0.03a	4.29e-08
Calcium (g/kg)		8.23 ± 0.55b	11.23 ± 0.21a	8.20 ± 0.1b	4.86e-05
Phosphorous (g/kg)		4.80 ± 0.1b	5.87 ± 0.06a	4.87 ± 0.25b	0.000435

Heavy metals	Standard limit value				

Iron (mg/kg)	250^4^	45.02 ± 0.01c	91.93 ± 0.17a	52.04 ± 0.01b	8.81e-15
Copper (μg/kg)	6000^5^	114.12 ± 0.2b	127.36 ± 0.06a	126.91 ± 0.07ab	2.01e-11
Zinc (mg/kg)	100^5^	56.11 ± 0.13c	71.36 ± 1.17b	93.05 ± 1.07a	1.43e-08
Cadmium (mg/kg)	1^4^	0.12 ± 0.02c	2.19 ± 0.05a	1.04 ± 0.082b	2.58e-08
Lead (mg/kg)	5^4^	ND^6^	0.011 ± 0.00	ND^6^	

Averages followed by different letters differ significantly at the 5% level.

Abbreviations: Az_diet, Azolla meal as the main source of protein; BSF, black soldier fly; BSF_diet, BSF larva meal as the main source of protein; SM_diet, soybean meal as the main source of protein.

1 Soybean meal as the main source of protein.

2 Azolla meal as the main source of protein.

3 BSF larva meal as the main source of protein.

4 ARSO, African Organization for Standardization [37].

5 EC, European Commission [38].

6 Not detected.

#### Impact of Azolla and BSFL meals on in vivo crude protein digestibility of formulated rabbit diets

**FIGURE 4:**
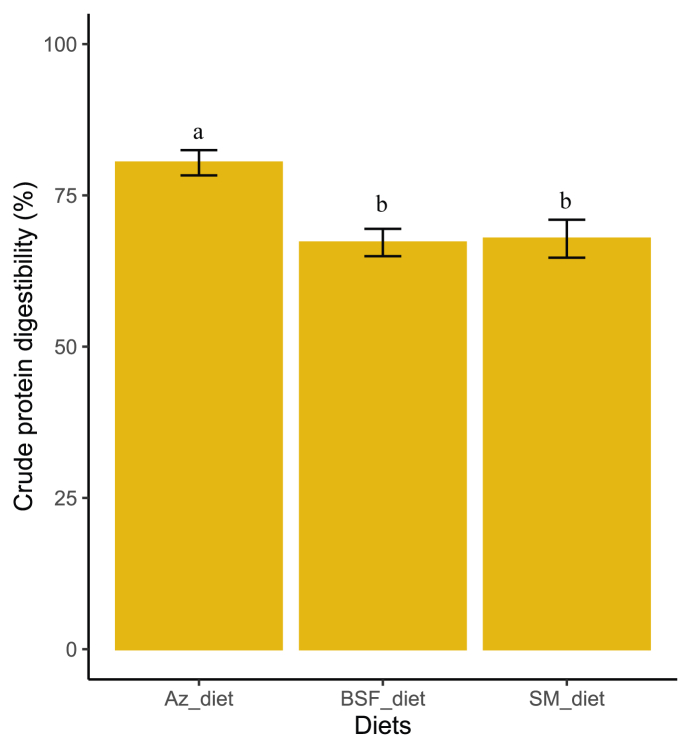
Crude protein digestibility of the formulated rabbit diets. Az_diet (Azolla meal as the main source of protein), BSF_diet (BSF larva meal as the main source of protein), and SM_diet (soybean meal as the main source of protein).

The CDP of the 3 experimental diets in rabbits is presented in Figure 4. The diet formulated with *Azolla spp.* (Az_diet) had the highest protein digestibility (80.39 ± 2.08%; *P* = 0.001187). The CDP of the control diet (SM_diet) and the diet formulated with BSFL (BSF_diet) was 67.83 ± 3.13% and 67.20 ± 2.26%, respectively. The CDP of the control diet was not significantly different from that of the diet formulated with BSFL.

### Impact of Azolla and BSFL meals on growth performance of rabbits

**FIGURE 5:**
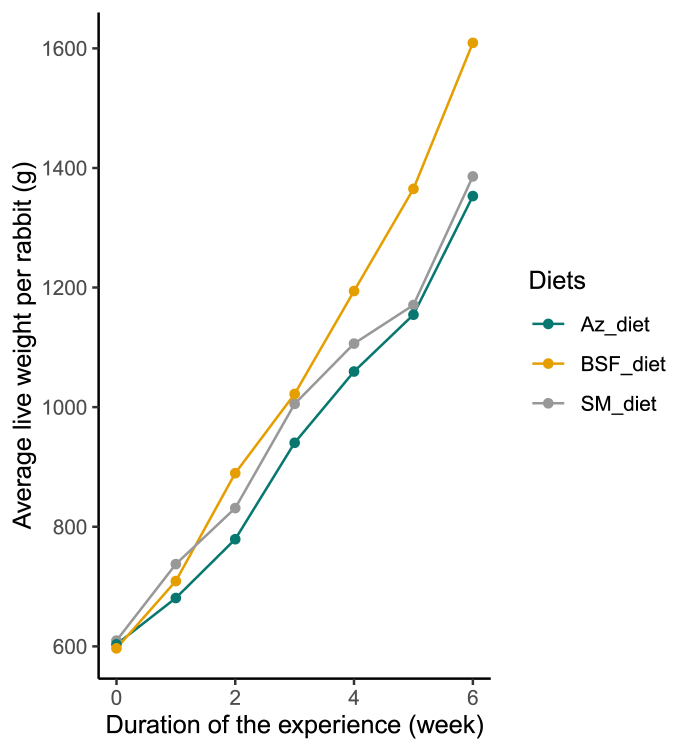
Weight growth of rabbits fed the formulated rabbit diets. Az_diet (Azolla meal as the main source of protein), BSF_diet (BSF larva meal as the main source of protein), and SM_diet (soybean meal as the main source of protein).

The growth parameters of rabbits fed the experimental diets are presented in Figure 5 and Table 3. On the basis of the curve, regardless of the diet, the rabbits' live weight increased gradually over time (Figure 5). However, rabbits fed the BSF_diet experienced statistically higher growth, reaching a final live weight of 1609.50 ± 113.71 g. The same was observed with the average weight gain of rabbits fed the BSF_diet, which was significantly higher (1012.5 ± 170.13 g) than that of rabbits fed the control diet (SM_diet) or the Az_diet. Also, the BSF_diet had the lowest feed conversion ratio of 2.80 ± 0.49 (Table 3). No mortality was recorded during the trial for any of the 3 treatments.

**TABLE 3:** Growth parameters of rabbits fed the formulated diets

Parameters	SM_diet^1^	Az_diet^2^	BSF_diet^3^	*P* value
Initial live weight (g)	609.58 ± 154.18	603.33 ± 101.70	596.67 ± 77.38	0.3467
Final live weight (g)	1385.83 ± 183.29b	1352.92 ± 131.54b	1609.50 ± 113.71a	0.000223
Final weight gain (g)	776.25 ± 84.35b	749.58 ± 114.13b	1012.5 ± 170.13a	1.93e-05
Feed intake (g)	2766.17 ± 28.18	2772.33 ± 25.28	2783.88 ± 38.05	0,7284
Feed conversion	3.60 ± 0.39a	3.78 ± 0.59a	2.80 ± 0.49b	8.55e-05
Mortality rate (%)	0.00 ± 0.00	0.00 ± 0.00	0.00 ± 0.00	—

Averages followed by different letters differ significantly at the 5% level.

Abbreviations: Az_diet, Azolla meal as the main source of protein; BSF, black soldier fly; BSF_diet, BSF larva meal as the main source of protein; SM_diet, soybean meal as the main source of protein.

1 Soybean meal as the main source of protein.

2 Azolla meal as the main source of protein.

3 BSF larva meal as the main source of protein.

### Impact of Azolla and BSFL meals on rabbit meat quality

#### Impact of Azolla and BSFL meals on carcass characteristics of rabbits

Information on rabbit carcass characteristics is presented in Table 4. The results indicated that the highest whole carcass yield was obtained with rabbits fed the BSF_diet. However, after evisceration, the highest gutted carcass yield was obtained with rabbits fed the Az_diet. There was no statistical difference in gutted carcass yields recorded with rabbits fed the control feed (SM_diet) and those fed BSF_diet. However, no significant differences were observed in liver and kidney yields (*P* > 0.05) for the 3 experimental treatments.

**TABLE 4:** Carcass characteristics of rabbits fed the formulated diets

Parameters	SM_diet^1^	Az_diet^2^	BSF_diet^3^	
Live weight (g)	1590 ± 60.83	1463.33 ± 132.04	1659.67 ± 274.74	
Whole carcass weight (g)	1259 ± 62.55	1106.67 ± 114.55	1311 ± 232.23	
Weight of viscera (g)	425.77 ± 43.80	485.47 ± 10.50	476.20 ± 66.25	
Gutted carcass weight (g)	699.43 ± 51.40	787.53 ± 45.39	796.63 ± 127.11	
Liver weight (g)	52.37 ± 4.94	53.83 ± 11.94	47.40 ± 1.71	
Kidney weight (g)	12.07 ± 1.10	12.87 ± 1.33	14.47 ± 5.46	

Yield (%)				*P* value

Whole carcass	79.16 ± 0.98a	75.56 ± 1.15b	78.92 ± 1.96a	0.038
Viscera	26.74 ± 1.96	33.35 ± 3	28.85 ± 2.86	0.0553
Gutted carcass	43.95 ± 1.59c	53.93 ± 1.84a	48,03 ± 0,55b	0.000438
Liver	3.29 ± 0.20	3.66 ± 0.59	2.89 ± 0.35	0.152
Kidney	0.75 ± 0.09	0.89 ± 0.17	0.86 ± 0.25	0.671

Averages followed by different letters differ significantly at the 5% level.

Abbreviations: Az_diet, Azolla meal as the main source of protein; BSF, black soldier fly; BSF_diet, BSF larva meal as the main source of protein; SM_diet, soybean meal as the main source of protein.

1 Soybean meal as the main source of protein.

2 Azolla meal as the main source of protein.

3 BSF larva meal as the main source of protein.

#### Impact of Azolla and BSFL meals on nutritional composition and heavy metal content of rabbit meat

Macronutrient analysis of rabbit meat revealed no significant differences in DM, lipid, and protein content between the 3 treatments. Mineral analysis revealed a very low accumulation of the targeted minerals in rabbit meat compared with initial dietary levels. Regardless of the diet tested in this study, the order of essential minerals in rabbit meat, according to their concentrations, was: P > Zn > Cu > Fe (Table 5). The highest Cu content was found in meat from rabbits fed the Az_diet, and the highest Zn content was obtained from rabbits fed the BSF_diet. No significant differences were observed in the P or Fe content of the meat from the 3 treatments. Pb and Cd were not detected in rabbit meat under any treatment.

**TABLE 5:** Nutritional composition and heavy metal content of rabbit meat expressed on a dry matter basis

Nutritional composition	SM_diet^1^	Az_diet^2^	BSF_diet^3^	*P* value
Dry matter (%)	23.33 ± 1.15	25.33 ± 0.57	25.33 ± 1.15	0.137
Crude protein (%)	20.59 ± 0.07	20.54 ± 0.13	20.60 ± 0.02	0.6703
Crude fat (%)	2.09 ± 0.03	2.11 ± 0.06	2.17 ± 0.02	0.1052
Phosphorous (mg/kg)	270.56 ± 1.79	272.51 ± 2.4	270.15 ± 0.37	0.4035
Heavy metals				
Iron (mg/kg)	0.01 ± 0.00	0.01 ± 0.00	0.01 ± 0.00	—
Copper (mg/kg)	1.54 ± 0.08b	3.24 ± 0.15a	2.96 ± 0.04ab	1.69e-06
Zinc (mg/kg)	12.86 ± 0.03c	17.68 ± 0.15b	27.48 ± 0.2a	5.43e-11
Cadmium (mg/kg)	ND^4^	ND^4^	ND^4^	—
Lead (mg/kg)	ND^4^	ND^4^	ND^4^	—

Averages followed by different letters differ significantly at the 5% level.

Abbreviations: Az_diet, Azolla meal as the main source of protein; BSF, black soldier fly; BSF_diet, BSF larva meal as the main source of protein; SM_diet, soybean meal as the main source of protein; ND, not detected.

1 Soybean meal as the main source of protein.

2 Azolla meal as the main source of protein.

3 BSF larva meal as the main source of protein.

4 Not detected.

## Discussion

Good-quality feed should meet all an animal's nutritional requirements, be palatable, digestible, and healthy. When introducing any new ingredient to a feed formula, it is essential to ensure the quality of the final product. This study aimed to evaluate the influence of Azolla and BSFL meals as new protein ingredients on the palatability and digestibility of formulated rabbit pellets, as well as on the nutritional quality and safety of rabbit meat.

### Impact of Azolla and BSFL meals on the quality of formulated rabbit diets

The feed preference test involved the following 5 feeds: Az_diet (Azolla meal as the main protein source), BSF_diet (BSF larvae meal as the main protein source), SM_diet (soybean meal as the main protein source), BSFL (live BSF larvae), and AZL (fresh *Azolla spp.* leaves). The aim was to observe how rabbits would react to these new ingredients in their raw form and in a feed formula. The results revealed that, regardless of their position in the test, the pelleted diets (Az_diet, BSF_diet, and SM_diet) were the rabbits' first choice and the ones they consumed most (FIGURE 2, FIGURE 3). Therefore, the rabbits studied were more attracted to pelleted feeds than to fresh AZL or live BSFL. This shows that the feeding behavior of rabbits can be influenced by how the feed is presented. Previous studies have also confirmed this observation. Mahamadou et al. [39] demonstrated that, in a free-choice situation, rabbits preferred pelleted feed to mash by 97%. Kpodékon et al. [40,41] also reported that rabbits clearly preferred pellets to forage.

Of the 3 feeds presented in pellet form, the SM_diet was consumed most widely. It was therefore more palatable to the rabbits in this study. The amount of feed consumed by a rabbit often depends on its palatability and energy value [42]. Increasing the digestible energy content of a feed reduces consumption by the rabbit. This is because rabbit’s appetite is regulated by a chemostatic mechanism, meaning that their daily energy intake remains constant [43]. Therefore, to meet their daily energy requirements, rabbits depend to some extent on the digestible energy content of their feed [44]. However, the 3 pellets offered were isocaloric (Table 2). In this case, therefore, it seems that the feed preference of the studied rabbits studied is more motivated by the immediate palatability of the feed than by its energy value. It is well known that rabbits prefer sweet flavors [45]. The sweet flavor of feed can come from the amino acids and monosaccharides present in it. The amino acids responsible for the sweet flavor of food/feed are often alanine, glycine, and arginine [46]. Furthermore, glycine and alanine are powerful odors that can stimulate feeding behavior by increasing feed intake [47,48]. However, these amino acids were not measured in the 3 feeds tested. A more in-depth study of the content of amino acids, fatty acids, monosaccharides, and volatile compounds in these feeds would be necessary to better understand the observed feed preference in rabbits during this study.

Analysis of the chemical composition of the 3 formulated diets revealed that, with regard to essential amino acids, the substitution of soybean meal with BSF meal increased the content of lysine and methionine (Table 2), as was observed by Oteri et al. [49] when fishmeal was substituted with BSF meal. However, substitution of soybean meal with Azolla meal resulted in a reduction in lysine and methionine content. In fact, methionine and lysine are among the most abundant amino acids in BSFL [50]. The methionine and lysine content of BSFL is higher than that of soybean meal [51,52]. Nevertheless, FAO data [53] indicate that lysine predominates in Azolla and that sulfur-containing amino acids do not meet the recommended value of 3.5 g/100 g of protein for animal feed.

High levels of Fe and Cu were observed in the Azolla meal formulation (Az_diet), whereas the BSF meal formulation (BSF_diet) had a higher Zn content than the control diet (SM_diet) (Table 2). However, the levels of these minerals detected are all below the recommended standard limit values for rabbit feeding [54]. Furthermore, these elements are essential minerals that play an important role in animal performance and meat quality. Zn improves growth, bone development, enzyme structure, organ function, and the immune system of animals [55,56]. Fe is required for blood and muscle pigments, and is actively involved in redox reactions. Cu is involved in enzymatic activities and oxygen transport [57,58]. The toxic elements analyzed were Cd and Pb. Cd was detected in all 3 diets (Table 2), with the elevated levels in the Az_diet and BSF_diet compared with the control diet (SM_diet). Pb was only detected in the diet containing Azolla meal (Az_diet). Therefore, substituting soybean meal with BSF and Azolla meals increased Cd and Pb levels in the diets. This could be explained by the ability of BSFL and Azolla plants to bioaccumulate heavy metals from their rearing substrate. Research has shown that Azolla plants can remove hazardous chemicals from water through a process called bioremediation [59]. This raises concerns about the use of Azolla in animal feed. Heavy metals can also accumulate in BSF at all developmental stages (larvae, pupae, and adults), with concentrations in larvae increasing in line with concentrations in the rearing substrate [11]. Additionally, Moniello et al. [60] and Kim et al. [61] reported high concentrations of toxic elements in experimental diets in which soybean meal had been replaced with insect meal at inclusion rates ranging from 7.3% to 50%.

The apparent digestibility of crude protein in rabbits fed the BSF_diet was similar to that in rabbits fed the control diet (Figure 4). This is contrary to the results of Volek et al. [36]. In their study, including cricket (*Acheta domesticus*) and yellow mealworm (*Tenebrio molitor*) meals in the diet decreased protein digestibility compared with a diet soybean meal-based diet. In our study, the apparent digestibility of crude protein was lower in rabbits fed the BSF_diet than in those fed the Az_diet. The observed decrease in digestibility in rabbits fed the BSF_diet could be due to the presence of chitin in BSFL. BSFL have a chitin content of ≤7% on a DM basis [62]. It should be noted that rabbits cannot digest chitin [3]. This is because herbivorous animals like rabbits do not possess functional acid chitinase genes and are therefore unable to digest chitin. In contrast, Azolla is easily digestible by rabbits because of its low lignin content [63]. This explains the protein digestibility achieved in rabbits fed the Az_diet.

### Impact of Azolla and BSFL meals on growth performance of rabbits

There was no significant difference in the average amount of feed consumed by each rabbit across the control diet (SM_diet), the Az_diet, and the BSF_diet (Table 3). This suggests that a pleasant smell or taste of the feed is not essential for regulating feed intake in rabbits. Regardless of the diet, there was no mortality and the rabbits showed good growth (Figure 4; Table 3). Also, the low average live weight recorded in this study (1352.92 ± 131.54 g) was within the normal range for growing rabbits of average breeds [64,65]. This indicates that all the rations contained adequate nutrients for the growth of captive rabbits. In terms of weight gain, rabbits fed the BSF_diet had significantly higher average weight gains (1012.5 ± 170.13 g). It is known that the quantity and quality of proteins, as well as the energy value, in an animal's diet influence weight gain. Because the 3 diets were iso-nitrogenous, iso-lipidic, and iso-energetic (Table 2), the difference in weight gain could be attributed to the quality of the proteins, specifically the higher lysine content of the BSF_diet (Table 2). Lysine, sulfur amino acids (methionine and cysteine), and threonine are considered the 3 most important amino acids for good growth in rabbits and must be provided by the diet [66,67]. The optimal requirements for lysine and methionine to promote good growth in rabbits are 7.5 g/kg and 3.5% of DM in the diet, respectively [68,69]. Furthermore, a study on New Zealand White rabbits found that decreasing dietary methionine and lysine levels reduced body weight gain and feed consumption [70].

### Impact of Azolla and BSFL meals on rabbit meat quality

The protein content of rabbit hind leg meat from the 3 treatments was similar to the value reported by Ajayi et al. [27] for rabbit meat produced in Nigeria: 20.78% DM. These values were also similar to the crude protein content of 20.8% DM for rabbit meat reported by the USDA (1963). However, the protein content in our study was slightly lower than that reported by Kowalska et al. [71], ranging from 21.85% to 22.10% for hind leg meat from rabbits fed diets containing silkworm pupae and mealworm larvae. The intramuscular fat content of hind leg meat from rabbits in the 3 analyzed treatments in this study is similar to that reported by several authors [65,71]. However, it is lower than the fat content (10.2% DM) reported by the USDA (1963) for rabbit meat. These differences could be due to variations in diet, because the nutrient content of meat is affected by the composition, of the animal’s diet, its age at kill, and its kill weight.

The annual global per capita consumption of rabbit meat is estimated at 0.19 kg [64]. The recommended daily intake of minerals such as Zn, Cu, and Fe for an adult human is 12.7 mg, 1.6 mg, and 6 mg, respectively [[72], [73], [74]]. Considering the average content of these micronutrients in the hind leg meat of rabbits from the 3 treatments, the annual global consumption of rabbit meat, as well as the absorption level of these minerals by the human body (a few percent), it can be concluded that they do not pose a risk to consumer health. However, high levels of Cu and Zn were noted in the hind leg meat of rabbits from the Az_diet and BSF_diet treatments, respectively. This indicates a possible accumulation of these minerals in rabbit meat. The levels of these minerals should therefore be monitored in rabbit feed. Studies on the contamination of foods of animal origin have found that, among toxic minerals (mercury, arsenic, Pb, and Cd), Cd and Pb pose the greatest risk to human health in terms of both the number of exceedances of permitted levels and the magnitude of the risk [24,75]. However, neither Cd nor Pb was detected in rabbit hind leg meat fed with the 3 diets. The nutritional and chemical composition of the hind leg meat in our study indicates that substituting soybean meal with BSFL meal and Azolla meal did not adversely affect rabbit meat quality.

## Conclusion

In a free-choice situation, rabbits preferred the control diet (formulated with soybean meal) to diets formulated with Azolla or BSFL meals. However, *Azolla spp* and *Hermetia illucens* larvae meals did not negatively affect growth performance and protein digestibility in rabbits in this study. Moreover, the inclusion of Azolla meal in the rabbit diet improved protein digestibility. Furthermore, toxic minerals such as Pb and Cd were not detected in the hind leg meat analyzed. Our results indicate that substituting soybean meal with BSFL meal and Azolla meal did not adversely affect rabbit meat quality. On the basis of the findings of this study, we can conclude that *Azolla spp* and *Hermetia illucens* larvae meals are suitable substitutes for soybean meal in rabbit diets. Future studies should consider the effect of these 2 alternative proteins on the chemical and organoleptic characteristics of rabbit meat.

## Author contributions

The authors’ responsibilities were as follows – EAO, CS, LMS, DD, JGB, RD: designed the study; EAO, AB, LMS: conducted the research; EAO: analyzed the data, performed statistical analysis, and wrote original draft; EAO, JGB, RD: wrote the manuscript and hold primary responsibility for the final content; RD: project administration, funding acquisition; and all authors: read and approved the final manuscript. IITA BENIN facilitated this research and provided the necessary expertise, setting, and all material needed for it.

## Funding

The authors reported no funding received for this study.

## Conflict of interest

The authors report no conflicts of interest.
